# Geodiversity for science and society

**DOI:** 10.1098/rsta.2023.0062

**Published:** 2024-04-01

**Authors:** Joseph Bailey, Richard Field, Derk van Ree, Franziska Schrodt

**Affiliations:** ^1^ School of Life Sciences, Anglia Ruskin University, Cambridge, UK; ^2^ School of Geography, University of Nottingham, Nottingham, UK; ^3^ Geo-engineering, Deltares, Delft, The Netherlands

‘Geodiversity’ defines the diversity of Earth's surface and subsurface, comprising features and processes across geology (including soil), hydrology and geomorphology (i.e. geodiversity elements). Geodiversity underpins the systems that support the living natural world: it is the steadfast, resolute partnership of humans and biodiversity, providing what the planet needs to support life and many materials we rely on in our daily lives. Its value is also inherent, with geoheritage sites enjoyed across the world. Humans have always had a close cultural connection with geodiversity and its elements, and its important role was highlighted in the 1990s in relation to ‘geoindicators’ [1]. Yet it is only recently that the importance of considering geodiversity has been more widely and deeply discussed. This is not least because of emerging unsustainable human and natural changes that threaten geodiversity globally. While climate and biodiversity are now routinely included in international conventions and policy briefings (e.g. in the form of ‘essential variables’), abiotic variables other than climate have been largely overlooked. This risks biased or inadequate policy and management decisions around surface and subsurface features and resources, as well as incomplete ecological analyses that may impair biodiversity conservation.

To move these discussions forwards, we applied for Royal Society Hooke Theo Murphy funding for a meeting of international experts across several facets of geodiversity research. We were awarded the funding in 2019, hoping for a spring 2020 meeting, but we all know what happened then. Instead of transferring the meeting online, we decided to wait until we could see each other in person. We were finally able to meet in January 2023 at the University of Nottingham, UK.

Publication of our PNAS paper on Essential Geodiversity Variables (EGVs; [2]) laid the foundation for this meeting. The paper was predicated on the idea that despite the importance of and threats to geodiversity, we do not have sufficient information to assess changes in geodiversity and the services it provides to us and nature at local, national and global scales. This is because: geodiversity tends to be accounted for differently (if at all) in ecological studies; there is no guidance on which elements of geodiversity are relevant for this purpose; and geodiversity data are not generally shared internationally in line with FAIR (findable, accessible, interoperable [being able to readily exchange information] and reusable) data principles. In response, we suggested learning from other disciplines to tackle this challenge, particularly work related to ‘Essential Variable’ (EV) frameworks. EV frameworks aim to align approaches in data collection, analysis and storage for effective and consistent monitoring of key planetary systems for the public, scientists and policymakers. They already existed for biodiversity, climate, oceans and sustainable development goals: we added geodiversity. After some progress since that publication, our meeting and this associated themed issue provide an update on novel research and remaining challenges on various facets of geodiversity discussions, with Schrodt *et al*.'s [3] paper giving an update on EGVs and what we need to do next.

All papers in this themed issue have had to in some way define ‘geodiversity’. The editors were not prescriptive about this because different fields approach the term in a slightly different way. The key is that the definition is clear in a given piece of work, and acknowledges if and where it differs from more widely used definitions. Widely accepted definitions encompass explicit surface and subsurface features and processes relating to specific geomorphological landforms and processes, geological types and hydrological features, for example, while broader definitions may also encompass topographic variables (e.g. elevation), and the broadest consider geodiversity analogous to the geosphere (i.e. lithosphere, atmosphere, hydrosphere and cryosphere). In this themed issue, Hjort *et al*. [4] in particular aim to make strides ‘Towards a taxonomy of geodiversity’, considering a language that everyone can rally around, drawing inspiration from the taxonomy we use to define living organisms.

We wanted to give readers a sense of the breadth of geodiversity in this themed issue, so Gray's [5] paper provides 10 case studies across different themes to give readers who are and are not well versed in geodiversity alike the chance to appreciate its geographic, practical and conceptual scope. All of the papers relate to sustainability in some way, but the contribution by van Ree *et al*. [6] tackles the topic directly, in relation to urban subsurface exploitation and its link to human wellbeing, highlighting insufficient frameworks for subsurface development and use, and suggesting ways forward.

Elsewhere in the themed issue, you will see papers on the measurement and monitoring of geodiversity and biodiversity, and conservation. This includes presentation of geodiversity data for Europe at two spatial resolutions [7]; development of a framework for quantifying geodiversity at local scales [8]; integrating vegetation and geodiversity monitoring using remote sensing and traits [9]; and quantitatively assessing geodiversity uniqueness and applying this to biodiversity conservation [10]. Seijmonsbergen *et al*. [11] bridge geodiversity measurement and geoconservation by creating a global geodiversity map, which they use to assess the extent to which UNESCO Global Geoparks represent the different elements of geodiversity, finding some components are underrepresented. Such global geodiversity maps are also considered in the Amazon drainage basin, where no large-scale geodiversity assessment has previously taken place, with global maps being refined to ultimately define three principal regions within the basin [12].

Geodiversity–biodiversity relationships are addressed empirically in two papers. Gerstner *et al*. [13] highlight the importance of considering the resolution of geodiversity data for species distribution modelling. The validity of taking a Conserving Nature's Stage approach to biodiversity conservation is assessed by Miller *et al*. [14], who find that conserving geophysical characteristics as surrogate sites for biodiversity is an effective conservation approach. Elsewhere in a geoheritage and geoconservation context, Monge-Ganuzas *et al*. [15] describe geoconservation achievements within the International Union for the Conservation of Nature (IUCN) and offer further recommendations. Arid Quaternary geoheritage sites are specifically considered in the context of promoting inclusion and diversity as part of the assessment process [16], while the dilemma faced by many geoheritage sites of balancing protection with geotourism, including valuation of geodiversity, is tackled by Anougmar *et al*. [17]. Schrodt *et al*. [3] also provide an important update on EGVs and propose areas of focus to speed up development and further integration of EGVs across the scientific and policy communities.

Our intention was always for this themed issue to encourage engagement with EGVs going forward. To achieve this, we collated current knowledge to provide an important learning resource around geodiversity, critically assess progress, raise awareness of the importance of geodiversity generally and specifically EGVs, and chart a course for interdisciplinary discussions around the importance of the Earth's geodiversity for sustainability, including biodiversity and the economy. We hope you enjoy it, and we look forward to developing these themes further.

## Data Availability

This article has no additional data.
